# Electrically Tunable Nd:YAG waveguide laser based on Graphene

**DOI:** 10.1038/srep36785

**Published:** 2016-11-11

**Authors:** Linan Ma, Yang Tan, Shavkat Akhmadaliev, Shengqiang Zhou, Feng Chen

**Affiliations:** 1 School of Physics, State Key Laboratory of Crystal Materials and Key Laboratory of Particle Physics and Particle Irradiation (Ministry of Education) Shandong University Shandong, Jinan, 250100, China; 2 Helmholtz-Zentrum Dresden-Rossendorf, Institute of Ion Beam and Materials Research, Bautzner Landstrasse 400, 01328 Dresden, Germany

## Abstract

We demonstrate a tunable hybrid Graphene-Nd:YAG cladding waveguide laser exploiting the electro-optic and the Joule heating effects of Graphene. A cladding Nd:YAG waveguide was fabricated by the ion irradiation. The multi-layer graphene were transferred onto the waveguide surface as the saturable absorber to get the Q-switched pulsed laser oscillation in the waveguide. Composing with appropriate electrodes, graphene based capacitance and heater were formed on the surface of the Nd:YAG waveguide. Through electrical control of graphene, the state of the hybrid waveguide laser was turned on or off. And the laser operation of the hybrid waveguide was electrically tuned between the continuous wave laser and the nanosecond pulsed laser.

Graphene, as a two-dimensional material, has unique electrical and optical properties^1^,^2^,^3^. In optics, graphene has uniform linear and nonlinear optical properties over a broad spectral range, enabling the discovery of novel photonic devices. For example, graphene exhibits a strong polarization-dependent absorption integrating with fiber or waveguide devices^3^. And, graphene can be used as an efficient saturable absorber (SA) in a laser system for the generation of ultrafast lasers^4^,^5^,^6^,^7^. Another fascinating feature is that the Fermi level of a graphene layer can be tuned by electrical signals^4^,^8^,^9^,^10^,^11^,^12^,^13^. A shift in the Fermi level will change the optical absorption of graphene, allowing the electrical control of the optical property of graphene. Besides, graphene has the Joule heating effect and a high intrinsic thermal conductivity^14^,^15^, which is attractive for various thermal applications including the transparent heater with fast reaction and conductors.

Waveguide laser is one of key elements for photonic circuits, integrated by the waveguide structure and other micro-optic elements^16^,^17^. The waveguide structure acts as the laser resonant cavity and allows a low laser threshold and a high slope efficiency, because of the high degree of light confinement in the waveguide architecture. Combining with graphene, the output of the waveguide laser can be changed to the Q-switched laser pulses, due to the saturable absorption of graphene. There are two ways to integrate graphene with the waveguide. One is coating graphene onto the output facet of the waveguide, and the guided light passes through the graphene sheet^6^. The other one is covering the waveguide with the graphene sheet, the graphene sheet absorbs the guided light via the evanescent field of the waveguide^18^. Recently, it is reported that the evanescent field absorption may guarantee the maximum efficiency of the nonlinear effect of the layered materials in the fiber^19^,^20^. And the evanescent field absorption of graphene can be conveniently tuned by the electrical signals in the fiber laser system^12^,^13^. According to reports about the evanescent field absorption in the fiber laser, we believe the electrical controlled laser can also be obtained based on the waveguide, via the controllable evanescent field absorption. Recently, we note that a kind of carbon-nanotube-based laser is independent of the polarization of pulses and has highly environmental stability^21^,^22^. Especially, a distributed ultrafast laser is reported firstly by Liu *et al*.^22^. However, the carbon nanotube is not tuned electrically.

In this work, we demonstrate an electrically controllable hybrid Graphene - Nd:YAG (neodymium doped yttrium aluminum garnet) cladding waveguide laser. Multi-layer (4 layers) graphene and electrodes were coated onto the surface of a Nd:YAG cladding waveguide, constituting a graphene-based capacitance or an electric heater (depending on the connection of the electrical circuit). Under the pumping of 810 nm laser, the laser emission was obtained in this hybrid waveguide. Tuning electrical signals of the capacitance, the laser operation of the hybrid waveguide was tuned by electrical signals from continuous wave (CW) to Q-switched regime (40 ns). Switching the electrical circuit to the electrical heater, the “on or off” state of the waveguide laser was also electrically controlled.

## Results and Discussion

### Linear absorption

**Figure 1:**
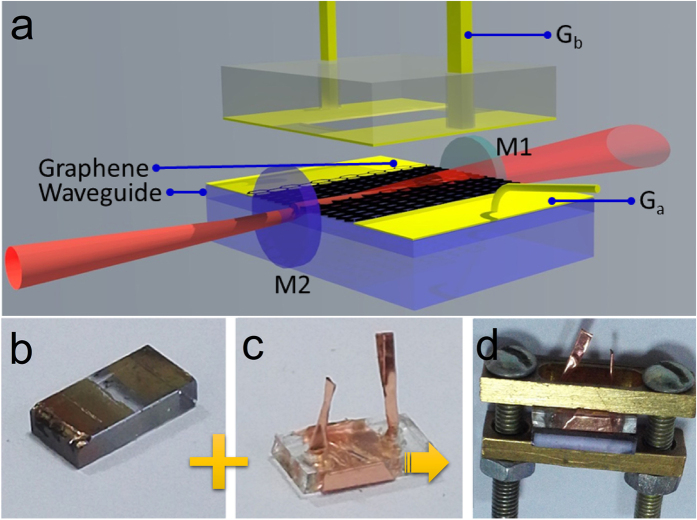
Electrically controlled hybrid graphene-Nd:YAG waveguide. (**a**) Schematic diagram of electrically controlled hybrid graphene-Nd:YAG waveguide and the experimental setup for the waveguide laser. Photographs of the Nd:YAG waveguide (**b**), quartz (**c**), and combined structure (**d**).

Figure 1(a) shows the schematic diagram of the electrically controlled hybrid graphene-Nd:YAG waveguide. An electrode (G_a_) was deposited onto the waveguide surface with a gap of 1 mm (Fig. 1(b)). The multilayer (four layers) graphene covered the gap on the G_a_. Another (G_b_) electrode was coated onto the largest facet (5 × 10 mm^2^) of quartz with a dimension of 5 × 7 × 3 mm^3^, leaving a tiny gap (0.5 mm) (Fig. 1(c)). The tiny gap has a overlap with the interval space in G_a_, and it was used to avoid the absorption of the guided light by the electrode. By pressing the quartz onto the surface of the waveguide, two electrodes (G_a_ and G_b_) constituted a capacitance (Fig. 1(d)). A refractive-index-matching liquid (~1.52) was dripped between electrodes, to prevent the oxidation of graphene.

**Figure 2:**
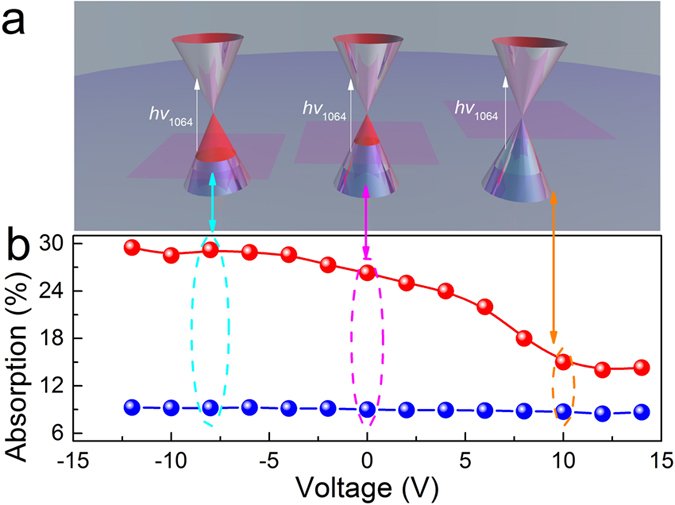
Linear absorption of the hybrid waveguide. (**a**) Shift of the Fermi level of graphene. (**b**) Variation in the linear absorption of the hybrid waveguide as a function of voltage.

The linear absorption of the hybrid graphene–Nd:YAG waveguide was measured at the wavelength of 1064 nm with a power of 1 mW. Without the electric signal, the absorption ratio of the hybrid waveguide was ∼26.3% and 9%, corresponding to the polarization parallel (*p*) and vertical (*s*) to the graphene film. A graphene-based capacitance was built by connecting *G*_a_ and *G*_b_ into the electric circuit. Pink arrows in Fig. 1(a) shows the direction of the current. The position of the Fermi level of graphene can be tuned by applying a drive voltage signal to the capacitance, determining the optical absorption of graphene (Fig. 2(a)). In the positive voltage region, charges accumulated in the graphene film, increasing the Fermi level near the Dirac point. This allowed the excitation of more electrons by light, inducing the ehance linear absorption of graphene. However, in the negative voltage region, the Fermi level decreased because of the lack of electrons, which blocks the optical absorption of graphene. The electrically controlled linear absorption of hybrid waveguide is shown in Fig. 2(b). With *p*-polarization, the absorption ratio increased to 29.2% at −8 V and decreased to 15% at 15 V. Obviously, the absorption of graphene was electrically controlled. For *s*-polarization, the variation was from 9.2% to 10%, which is not significant.

### Q-switched waveguide laser

**Figure 3:**
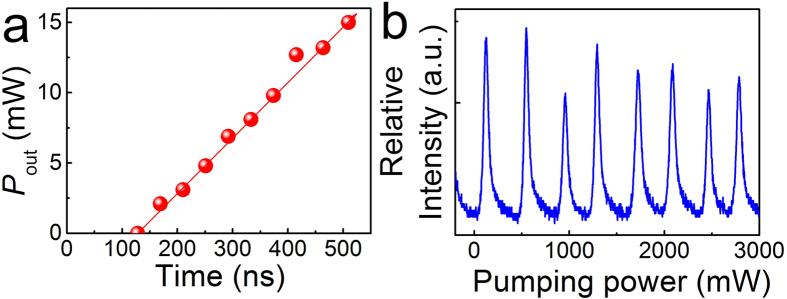
Q-switched waveguide laser. (**a**) Average output power and pulse energy vs. launched pump power. (**b**) Pulse profile of the waveguide laser for a pump power of 500 mW.

Graphene worked as a saturable absorber in the hybrid graphene-Nd:YAG waveguide laser. The laser performance of the hybrid waveguide without electrical signals is shown in Fig. 3. Under the 810-nm pumping laser, the laser emission was obtained at a wavelength of 1064 nm. The laser threshold and slope efficiency were 126 mW and 4%, respectively. When the pumping power was above 160 mW, stable pulse trains of the output laser were observed. At a pumping power of 500 mW, the output power had the maximum value of 15 mW, and the pulse train corresponding to the pulse duration (repetition rate) of 40 ns (2 MHz) is shown in Fig. 3(b).

### Electrical tuning of the saturation absorption of graphene

**Figure 4:**
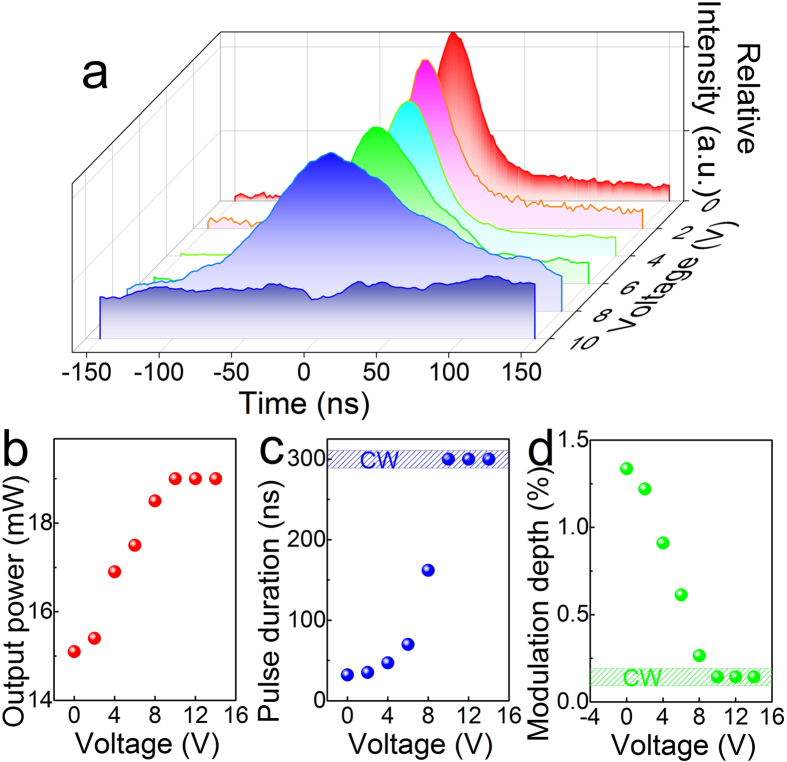
Electrical tuning of the saturation absorption of graphene. Variations in the pulse profile corresponding to the electric signal from 0 V to 10 V. Electro-optical response of the hybrid graphene-Nd:YAG cladding waveguide laser. Variations in output power (**b**), pulse duration (**c**), and modulation depth of graphene (**d**) with voltage.

The nonlinear absorption of graphene corresponding to the electric signal was measured based on the variation in the pulse profile of the output laser. When *G*_a_ and *G*_b_ were connected to the electric circuit, a broadening of the pulse profile was observed with the increase in voltage from 0 V to 8 V. When the voltage was above 10 V, the pulse train completely disappeared, and the output light was a CW laser. The evolution of the switching from a pulse laser to a CW laser is shown in Fig. 4(a).

The gate-voltage-dependent laser performance of the hybrid waveguide is shown in Fig. 4. With the increasing of the voltage to 14 V, the power of the output laser slowly increased from 15 mW to 19 mW (Fig. 4(b)). The pulse duration was broadened from 32 ns to 260 ns (Fig. 4(c)) with the increase in voltage. According to the model for the passively Q-switched laser^23^,^24^, the equation for the modulation depth could be calculated using the following equation:
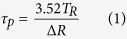
where Δ*R* is the modulation depth, *τ*_p_ is the pulse duration, and *T*_R_ is the cavity round-trip time. As shown in Fig. 4(d), the applied voltage reduced the nonlinear absorption of graphene, decreasing the modulation depth from 1.2% (nanosecond laser) to 0% (CW laser).

### On-off switcher of the waveguide laser

**Figure 5:**
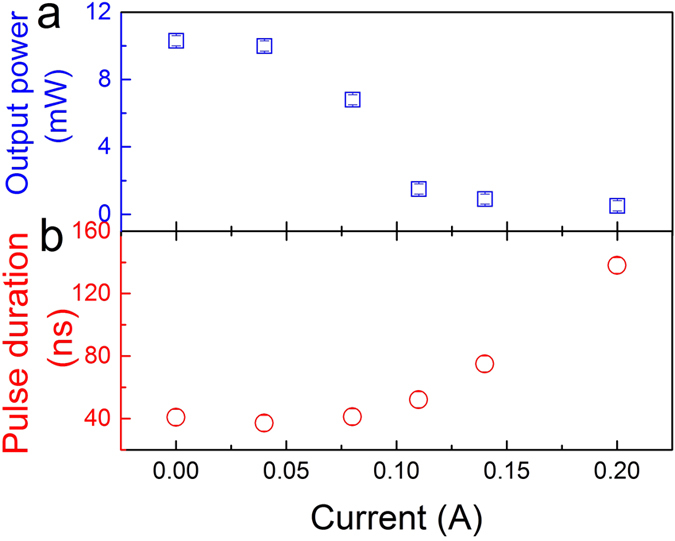
On-off switcher of the waveguide laser. (**a**) Experimental measured output power (square) of the hybrid waveguide as a function of the current. (**b**) Variation of the pulse duration (circle) along with the current.

Connecting the electrode *G*_a_ into the electric circuit, a graphene based transparent flexible heater was formed to thermally tuning the hybrid waveguide laser. The direction of the current was shown by purple arrows in Fig. 1(a). In this case, the resistance of graphene was 220 Ω and the electric current was tunable between 0 A and 0.2 A. The maximum temperature variation on the surface of the hybrid waveguide was measured to be 106 ± 10 K. Graphene based heater modulated the optical response of the hybrid waveguide in two ways. At first, heating treatment temporarily decreased the refractive index of the Nd:YAG crystal in the waveguide region due to its Joule heating effect of the Nd:YAG crystal, which affected the confinement of the waveguide structure to the guided light. Second, the excellent fluorescence of the Nd:YAG was weaken at the high temperature^25^,^26^. Through the combination of two effects, the laser emission from the hybrid waveguide can be turned off by graphene based heater. Figure 5(a) demonstrate the variation of the output laser along with the current. In the low current (below 0.1 A), the output power was slowly decreased from 10 mW to 7 mW with the current increasing and the pulse duration was kept at ~40 ns corresponding to the modulation depth of 1.1% (Fig. 5(b)). Above 0.1 A, the output power quickly dropped to zero, and the response time was less than 0.1 s. Although the response time of this setup is too long to realize the ultrafast control of the on-off states of the waveguide laser. We believe this work still has the application potential for the electrical switcher of the waveguide laser, which do not need a fast response.

## Discussion

This work demonstrates a novel way to control the waveguide laser emission via electrical signals. Multilayer graphene was coated onto the surface of the Nd:YAG waveguide, which composed a hybrid Nd:YAG waveguide. Due to the saturation absorption of graphene, the Q-switched pulsed laser was generated in the Nd:YAG waveguide under the 810 nm laser pumping. In order to control the optical property of graphene, electrodes were connected with graphene. Due to different ways of the connection, graphene displayed different functions.

Connecting graphene into a capacitance, the Fermi level of graphene is tuned by the electric signal. Low down the Fermi level far below the Dirac point, the optical absorption ratio of graphene could be decreased from 29.2% to 15%. Meanwhile, the modulation depth could be modulated between 1.2% and 0%. As a result, the output laser from the hybrid waveguide laser can be switched between CW and Q-switched laser by electric signals.

Connecting graphene into the electric circuit as a heater, the temperature of the hybrid waveguide could be modulated by graphene. The heating treatment temporarily decreased the refractive index of the Nd:YAG crystal in the waveguide region, which affected the confinement of the waveguide structure to the guided light. Besides, the fluorescence of the Nd:YAG was weaken. Therefore, the waveguide laser could be controlled between states of on and off.

This work realized the control of the waveguide laser between CW laser, Q-switched laser, on and off, via connecting graphene with electric circuits. It will provide help for the further development of the controllable waveguide laser.

## Methods

### Hybrid Graphene-Nd:YAG waveguide

The waveguide was fabricated by ion irradiation on the surface of a Nd:YAG crystal (doped by 1 at% Nd^3+^ ions). The Nd:YAG crystal was cut into dimensions of 5 × 10 × 1.5 mm^3^, and the facets were optically polished. One of the largest facets was irradiated twice by a carbon ion beam with the energy and fluence of (6 MeV + 15 MeV) and (1 × 10^15^ + 2 × 10^14^) ion/cm^2^, respectively. After the multiple irradiation, a cladding waveguide was formed near the surface of the Nd:YAG crystal^18^.

### Waveguide laser

Lasing in the hybrid waveguide was obtained under the pumping using a free-space coupling scheme (Fig. 1). A continuous-wave laser (CW laser) at 810 nm was used as the pumping source and coupled into the waveguide through a lens (focal length 20 mm). Mirrors with reflectivities of 90% (M1) and > 99.8% (M2) at ∼1064 nm were adhered onto the end-facets of the hybrid waveguide as the input and output mirrors, respectively, building a resonant cavity for laser oscillation. The output light from the waveguide was collected by a long work-distance microscope objective.

## Additional Information

**How to cite this article**: Ma, L. *et al*. Electrically Tunable Nd:YAG waveguide laser based on Graphene. *Sci. Rep.* **6**, 36785; doi: 10.1038/srep36785 (2016).

**Publisher’s note:** Springer Nature remains neutral with regard to jurisdictional claims in published maps and institutional affiliations.
